# Observations on Functional Disorders of the Spinal Cord, and Their Connexion with Hysterical, Nervous, and Other Diseases; Illustrated by Cases, Selected Chiefly from the Reports of the Pallas-Kenry and Currah Dispensaries

**Published:** 1830-04

**Authors:** Wm. Griffin, D. Griffin

**Affiliations:** Limerick.; Limerick.


### FUNCTIONAL DISORDERS OF THE SPINAL CORD.

*Observations on Functional Disorders of the Spinal Cord, and their Connexion with Hysterical, Nervous, and other Diseases; illustrated by Cases, selected chiefly from the Reports of the Pallas-Kenry and Currah Dispensaries.* By Wm. Griffin, M.D. and D. Griffin, M.R.C.S. Limerick.

(Continued from page 215.)

### *Respiratory System.*

There are no parts of the human frame so easily excited, or so sensitive to the existence of irritation, as the respiratory system, by which we would be understood to mean all those parts or organs whose actions are usually associated in the act of breathing, and not any assumption with respect to Mr. C. Bell's theory. This extreme excitability accounts for its derangement, to a greater or lesser degree, in the diseases of almost every organ of the body, and renders it more incumbent on us to understand clearly the symptoms which may be referred to mere disturbance of function.

The most frequent symptom of irritation of the cervical and upper dorsal portion of the spinal cord, is cough; and if there was no other fact to prove the importance of more strict inquiry into the effects of disturbed influence of nerves, than the manner in which this has been wholly overlooked by our best medical writers, it would be sufficient. It occurs in very many forms, and connected with a vast variety of symptoms: sometimes it is hard, dry, and constant, or it comes on at certain hours in the day in violent convulsive paroxysms, with or without fever; and in either of these characters, it will generally be found accompanying gastric and liver affections; sometimes uterine or dental irritation, or that produced by intestinal worms; it occasionally occurs like common cold, accompanied by slight oppression, and it is in this form it appears in the general run of spinal cases, but is seldom, as in the foregoing, attended by expectoration. Lastly, it may be met with, slight, short, and little troublesome, just as it occurs in tubercular phthisis, and often in connexion with such symptoms as lead to the supposition of the existence of that fatal disease.

Spinal tenderness may be found in all these cases; and, as has been so often stated, may keep up the symptoms, after the cause in which it originated has been removed. Mr. Burns seems to apprehend that, in young females, if the spinal affection is overlooked or neglected, consumption may be induced. That irritation at the trunk of the respiratory nerves may induce tubercular inflammation as readily as when acting on their minute fibrillæ in the bronchial membrane, there can be no possible doubt; but this can only be admitted with respect to cases in which a phthisical diathesis prevails. Numerous instances have occurred to us in which most harassing cough was kept up for months, and even years, by irritation of the cervical portion of the medulla, without inducing any more formidable disease. One case only we recollect to have terminated in fatal consumption, but this was very protracted, and all our entreaties could not induce the patient to persevere in attention to the upper part of the spine, although the most marked benefit, and at one time an interruption of the disorder for months, was procured by repeated blistering: she could not be persuaded that remedies applied to the neck were of avail for what she conceived an affection of lungs. Indeed, it seems very probable that, in many cases of hereditary phthisis, the disorder commences in this way, and might be often prevented by vigilant attention. Cough may always be excited in these irritations by pressure on the tender vertebræ, and sometimes convulsive fits, or hard barking, occurs from any unusual action of the diaphragm, or accidental excitement of the mucous membrane of the lungs. Thus a sudden fit of laughter, or of crying, may bring on coughing for hours, or it may be occasioned by a stimulant vapour, or by mere mental excitement.

XXV. A young lady, aged seventeen, of delicate frame, light coloured hair, and peculiarly fair skin, was attacked with cold, which left a short, slight cough behind it, not very troublesome, but occurring frequently in the day, and accompanied by slight oppression. The oppression was greatly increased on ascending the stairs, and there was then some palpitation. The cheek was always coloured with a pink spot, beautifully defined and bright, especially in the morning. The pulse was quick and readily excited, usually 120 in a minute; the tongue was whitish towards the middle and back part, and the papillæ elevated; she had tenderness at the lower part of the sternum, and often pain there on deep inspiration, and complained of general languor and weakness. There was tenderness of all the cervical, and of the four upper dorsal vertebræ; pressure on any one of which instantly brought on coughing.

The treatment in this instance consisted in the application of ten leeches, and long narrow blisters, often repeated, to the tender part of the spine. Minute alterative doses of the bluepill were also given, and a mixture composed of Infus. Rosæ, with Epsom Salts and Tincture of Digitalis. The most evident amendment followed the blistering: in proportion as the spinal tenderness abated, the pulmonic affection gave way, and she was quite well in a fortnight or three weeks, although the attack had existed for a considerable time previous.

We shall, in contrast with the foregoing, give the case of a young lady of the same age, in which the spinal affection was of a much severer and more obstinate character, and which yet suggested little apprehension as to the result, from the evident antiphthisical character of her conformation. In the former, one could not but be alarmed as to the possible result; while in that which we are about to relate, and in which there was beyond all measure more of complaint and suffering, no person of experience could hesitate in predicting a favorable termination.

XXVI. A lady, aged seventeen years, became affected with pain in the right side, great tenderness on pressure, sickness of stomach and feverishness. The two last symptoms were removed by purgatives, but the pain continued, varying its situation slightly from beneath the left breast to the margin of the ribs, and occasionally as low as the crista of the ileum; sometimes it changed altogether to the left side, or affected both at the same time, and was very soon attended by nervous lowness and oppression, with other hysterical symptoms. She was attacked also by continued headach and sickness of stomach, and eventually by dry, loud, harsh cough; pain and soreness of chest; frequent chilliness and inclination to shivering, with a feeling of languor and debility. There was some furring of the tongue in the morning; the appetite was bad, and the pulse frequent and irritable, usually 120 in a minute. The catamenia were regular. On examination of the spine, great tenderness of the second cervical was discovered; pressure there occasioning acute pain in the vertex and brow; pressure on the lower cervical and upper dorsal excited pain at the spot, and loud coughing; at the seventh or eighth, the same symptom, with pain of chest; and at the four last dorsal, as well as at the margin of the ribs, as far forward as the ensiform cartilage, there was extreme pain on pressure.

Leeches and repeated blistering at the tender point of the vertebral column were employed, with great temporary relief; but, when the severity of the symptoms abated at one part, it increased at another, as if there had been rather a transference than an actual removal of irritation. Thus, she sometimes complained most of the head and stomach; sometimes of the distressing cough, pain of chest, and oppression; sometimes of the back and sides. A considerable amendment took place during the use of mild purgatives, followed by tonics; and the cough seemed at last to yield to the Sulphate of Quinine.

All the symptoms recurred, however, in an aggravated form a few weeks afterwards, in consequence of some uneasiness of mind: the cough grew more distressing, the pain in the right side, which had never wholly left her, became worse, and the catamenia appeared every three weeks. A mild mercurial course was now resorted to, not so much from a conviction that the symptoms originated in an affection of liver, as that we had reason to attach some value to its use in pure cases of spinal irritation. She had not been rubbing the mercurial ointment a fortnight, when the cough completely subsided, although resisting every other remedy for so long a period; and the general health gradually improved. Occasional blistering was still made use of, with advantage, and the side was covered with a Belladonna plaster.

There was once again a recurrence of the symptoms, after an interval of four or five weeks, but they were of a mild character, and yielded, as in the first instance, to purgatives and tonics, with small doses of opium, and to stimulating liniments applied to the spine. This last remedy was of such obvious advantage, that she declared it did her more good than all that were made use of from the commencement. Her recovery was perfect.

The hard, barking cough, which Dr. Clarke describes as affecting young females, and yielding to sudden effusion of cold water, after foiling every other remedies, was, we should suppose, a mere symptom of spinal irritation. Tenderness of the vertebræ would have been discovered, had any examination been made: nor, indeed, can we imagine a disorder of any other nature likely to be so suddenly and so perfectly relieved. The cough remaining after the acute attack of hooping-cough is over, and which is said to depend upon habit, is also perhaps dependent on an irritable state of the cord.* Since the conjecture oc-

* The Tussis spasmodica, which Underwood describes as affecting infants, remaining dry and hoarse under the use of pectoral remedies, but soon relieved by opiates or cicuta, is evidently of this nature. Perhaps, the same may be inferred of the cough described by Dr. Gregory, of London, as curred to us, however, we have met with no instance to confirm it. In typhoid, inflammatory, and still more frequently in intermittent fevers, cough is often a symptom of nervous disorder, and especially disorder of the upper part of the spinal cord. It is the more necessary for the practitioner to have this continually impressed on his mind, as from its connexion in these cases with high febrile excitement, it may very readily lead him to imagine he has to contend with local inflammation.

Next in frequency to cough, and usually accompanying it, is oppression, which may occur in all its degrees, from slight dyspnœa up to the most terrific asthmatic paroxysm, as the irritation may chance to be more or less intense, or affect one set of nerves or another. When the superior or external respiratory are engaged, we have uneasy breathing and a feeling of atmospherical pressure, not perceptible while the healthy relation subsists between it and the muscular power. This is most distinctly experienced by persons slightly affected with hemiplegia: they have a sense of weight or pressure, and of obstruction to the free expansion of the lungs on the paralytic side. When the phrenic is affected, we have a sense of oppression, a difficulty of expiration, and laborious action of the external respiratory muscles. When the par vagum suffers, either the muscular coats of the minute bronchial tubes are affected, as in pure spasmodic asthma, or the nervous influence, the secreting process of the lungs, by which a change is produced in the blood, is interrupted, as in the nervous; and in both a distressing sense of suffocation is experienced. When the functional disturbance is confined to the superior laryngeal or the recurrents, we have spasmodic action of the oblique and transverse arytenoid muscles, or loss of voice, or hoarseness, or wheezing, or croupy breathing; which last is, perhaps, owing to some change in the state of the mucous secretions.

The first species of oppression is, in its slighter degrees, a very frequent one, and is usually present in those instances in which patients complain of a load on the chest. It shall be more particularly noticed, as well as the affection of the phrenic, in subsequent cases, where it formed a prominent symptom. The fact that, in many persons, the asthmatic paroxysm depends solely upon disturbance of the dependent on an irritable state of the mucous membrane, which he describes as not benefited by any remedies which he has been able to devise, except change of air.

nervous influence, is a necessary pathological inference, adopting, as we do, our views of functional disorder as general with respect to the nervous system;* and it is to this description of cases, perhaps solely, that galvanism, as suggested by Dr. W. Philip, is applicable. Of the advantages resulting from a very slight modification of the usual treatment, many instances have occurred to us.

XXVII. A young lady, of an asthmatic constitution, and whose habit was so susceptible that town air or a close room instantly occasioned dyspnœa with piping respiration, caught a severe cold, and was in consequence attacked with a violent paroxysm, attended with considerable fever. She was found with purple cheeks and lips, supported in the bed by pillows; the chest heaving; the muscles of respiration tense and labouring; the pulse was 120, small and compressible. On examination, slight tenderness was discovered at the two or three upper cervical vertebræ. Together with other remedies usual in asthmatic cases, a blister was applied to the neck, much to the surprise of the patient, who had been always before blistered on the chest. Perfect relief to all the symptoms, but especially the oppression, was obtained, and the paroxysm on the next night was scarce observable.

In a few weeks afterwards she had a return of the fit in a more violent degree, and applied two large blisters in succession to the chest, at her own counsel, without the slightest benefit. Her medical attendant was now sent for, and found her, if possible, in a much worse state than on the former occasion. A blister was again applied to the neck, and a mild diaphoretic mixture, with Hyosciamus, ordered. Though not effecting so complete a resolution of the paroxysm as in that instance, it produced a surprising mitigation of the disorder. The young lady and her friends were particularly struck with the obvious relief which the remedy procured.

It must be evident that leeching, blistering, or friction with liniments at the origin of the nerves, can only be of use in the special instance of nervous disturbance, and perhaps we might say generally, only where spinal tenderness is to be met with. The following are cases of irritation, where the oppression and cough were merely symptomatic of the general affection.

* We feel particular pleasure in referring to a very interesting chapter in the work of Laennec, on nervous and spasmodic asthma. It places the question of the dependence of a class of asthmatic cases on mere nervous disturbance beyond all disputation.

XXVIII. Kate C—, aged fifty-six, was ill three weeks with violent oppression, which usually seized her at night, after retiring to bed, and obliged her to sit up. It always commenced by a sensation as if cold water trickled down the crown of the head, and then palpitation of the heart supervened, with violent dyspnœa, and a feeling as if she was about to die. The fit, after continuing from half an hour to an hour, terminated by general perspiration and debility. She had no cough or expectoration, and the oppression was seldom distressing until night. There was great tenderness of all the cervical vertebræ, and of the five or six upper dorsal. Pressure at the sixth occasioned pain under the right breast.

XXIX. Ann Fitzgerald, aged thirty, a nurse, complained of hard, dry cough, with oppression; pain in the right eye and brow, aggravated much on coughing; general soreness of the scalp; pulse natural, tongue whitish. Her illness followed a severe cold, attended by inflamed tonsils and pain of chest. On examination, there was found great tenderness of all the cervical and the sixth or seventh dorsal vertebræ.

XXX. Catherine M'Mahon, aged fifty, complained of pain in the head, followed by a pustular eruption. The right side of the head was first affected, and afterwards the left in the same way. She had pain in the cardiac region, increased on inspiration, and attended by hard cough and oppression; the stomach was dyspeptic, and there was occasional vomiting. She felt no pain in the back, except on examination. Pressure at the first and second cervical vertebræ excited pain in the brow; at the eighth or ninth dorsal, severely in the stomach, with tendency to syncope; pressure at the last cervical or upper dorsal brought on the coughing, and took away the breath.

XXXI. Bridget Connel, aged forty-five, complained of pain and soreness at the pit of the stomach and right side, beneath the margin of the ribs, increased severely by pressure on the eighth dorsal vertebra; had fluttering at the heart, with great debility, and almost constant dyspnœa; obliged to sit up in the bed at night, in consequence of the oppression. Had pain across the brows and general soreness of the scalp, with thirst and loss of appetite; tongue little furred. Pressure behind the mastoid process brought on the pain at the brow, and there was considerable tenderness of the lower cervical vertebræ.

All these cases recovered. We have thought it unnecessary to occupy the reader's time with the treatment, as it is a subject upon which we shall hereafter dwell more particularly. It was necessarily modified by the variation in the symptoms, but was, through all of them, governed by the state of the spinal cord. Much as the symptoms seemed sometimes to indicate inflammatory action, bleeding was never resorted to, except with the view of allaying irritation, or relieving congestion, and then but to a small extent. It is remarkable how very often the stomach, and sometimes the liver, seem to be engaged in these cases, as in xxx. and xxxi. When it is recollected that these viscera are supplied by the same nerve which is distributed to the lungs, there seems little occasion to refer the connexion to any general sympathy, and we find a ready explanation of the fact adverted to by many medical writers, of the frequency with which the asthmatic paroxysm is occasioned by disordered stomach.

A very important fact, ascertained by Magendie and Desmoulins, is the constant correspondence between the action of the posterior portion of the fourth ventricle and the eighth pair. It is to this, perhaps, we are to refer the oppression and general affection of the respiratory system, which, in the commencement of apoplectic or paralytic attacks, we have sometimes seen mistaken for symptoms of hydrothorax. To this also we may attribute the occurrence of that very common affection, spasmodic croup, and one which has been said equally to resemble croup, described by Drs. Hamilton and Clarke, and which to us appears but a variety of the former.

That spasmodic croup is in most instances dependent either on irritation at the origin of the eighth pair, or at some part of the fourth ventricle, we need scarcely offer further proof than the frequency of its occurrence as a symptom of spinal irritation in adults. It is mentioned as one of that long train of affections under which the lady, whose case is first detailed in these papers, suffered, and shall be often noticed in others similarly complicated, as we proceed. It occurs sometimes like acute croup, with wheezing, as if from phlegm in the trachea, and stridulous respiration; sometimes with a peculiar crowing inspiration, as in the convulsive attack first described by Dr. Hamilton, of which we shall have to speak presently; and in these cases it is often combined with difficulty of swallowing, strongly marking its cerebral or spinal dependence. It is sometimes accompanied by feverishness, especially in children, in which case it is apt to be mistaken for true inflammatory croup; and it is generally induced by distant irritations, as teething or disordered bowels, or the accession of measles in certain unfavorable states of the atmosphere.

Three cases of this affection, occurring within a few days on the accession of measles, lately occurred to us, which sufficiently establish the foregoing views. In one, bleeding and vomiting was made use of, with immediate and perfect relief. As it was the first which occurred, and measles was not suspected at the time, it was supposed to be a case of true inflammatory croup. The occurrence of measles, however, on the next day, suggested its dependence on irritation: and the second little patient, who was quite as seriously attacked, was left to the warm bath, the action of an emetic, and calomel, which gave relief in the course of a few hours. In the third case, nothing was done, through some neglect on the part of the mother; but the child was nevertheless relieved in the morning by the eruption of the measles. What yet further proves the nature of these affections is, that, in the second case, after the infant had struggled through a severe and protracted disease, there was a recurrence of the croup, in as violent a degree as before. It was relieved by the same remedies, by blistering the neck, and by antispasmodics, and the child, though extremely exhausted, seemed likely to recover. It then, however, fell into frequent dozing; its eyelids were unclosed while asleep, and it was continually sawing the air with whichever arm chanced to lie out unconfined by the bedclothes. There was occasional grinding of the teeth, and other symptoms of an affection of the brain; the skin and feet were cold, and the face pallid. He remained in this state for a day or two, fell into convulsions, and died. There was no croupy breathing for the last few days. It would appear very probable that all these symptoms were rather the effect of exhaustion than of organic disease of the brain; but there was no examination permitted.

In so freely assuming the existence of spasmodic croup as a distinct disease from the inflammatory, it may perhaps be necessary to offer some remarks on the opinion of one who is justly esteemed of very high authority on this subject, Dr. Cheyne. He asserts that he has not been able to see any just grounds for considering that there are two kinds of croup, and that, from the identity of symptoms in what have been called the spurious and the inflammatory, he conceives them to be but varieties of the same complaint.

The pathological principles on which all our reasonings respecting functional disorders are founded, would, we think, without any reference to the immediate cases under consideration, lead to very opposite inferences: the functions of every organ in the body may be disturbed, as we have often insisted on, in three different ways; by inflammation immediately affecting any particular one, by irritation of the capillary extremities of the nerves distributed to it, by irritation at the trunk or origin of these nerves; and so like shall be the disturbance, so similar the symptoms in each of these cases, that sometimes no trace of distinction can be detected. Thus, ischuria may be occasioned by inflammation at the neck of the bladder, by the irritation of a stone within it, by irritation of the spinal cord at the origin of the lumbar nerves. Pain of the stomach or bowels may be occasioned by inflammation, by irritants acting on the minute nervous fibrils, or by irritation of the cord at the origin of some of the dorsal nerves; and in these latter cases the symptoms occasioned by mere irritation may be attended with heat of skin and quickness of pulse, as in the inflammatory. From all fair analogy, therefore, we must believe that the trachea may be affected with inflammation, (the inflammatory croup of authors;) with irritation, as from mephitic and irritating vapours acting immediately on the parts; and with irritation of the cord affecting the origins of the cervical and pneumogastric nerves: and that in all these the symptoms shall be precisely alike, as far as regards disturbance of function: they shall all be cases of croup, though arising from such different causes. So clear do these inferences appear to us, that, if the spurious or spasmodic croup was at this moment an unknown disease, we should unhesitatingly predict the probability of its occurrence.*

[To be continued.] 379.

* Little as the attention of the medical world seems to have been directed to the law just referred to, proofs of its universality are to be found in the history of the diseases of every organ of the human frame. Thus, as we have spurious croup, a mimic of the inflammatory, we have an affection of the larynx presenting a likeness of laryngitis, or of ulcerated larynx. Dr. G. Gregory mentions (Elements of the Theory and Practice of Physic,) that, "in the progress of consumption, particularly towards its latter stages, it is not unusual to find a violent pain come on, referred to the larynx, and attended generally with hoarseness. From the violence of the pain, it might be supposed owing to inflammation; but leeches and blisters are of no service, and it generally goes off in four or five days. It is probably a symptomatic pain, connected perhaps with the recurrent nerve."

